# Horizontal Plasmid Transfer Promotes the Dissemination of Asian Acute Hepatopancreatic Necrosis Disease and Provides a Novel Mechanism for Genetic Exchange and Environmental Adaptation

**DOI:** 10.1128/mSystems.00799-19

**Published:** 2020-03-17

**Authors:** Songzhe Fu, Dawei Wei, Qian Yang, Guosi Xie, Bo Pang, Yongjie Wang, Ruiting Lan, Qingyao Wang, Xuan Dong, Xiaojun Zhang, Jie Huang, Jie Feng, Ying Liu

**Affiliations:** a Key Laboratory of Environment Controlled Aquaculture (KLECA), Ministry of Education, Dalian, China; b College of Marine Science and Environment, Dalian Ocean University, Dalian, China; c Institute of Microbiology, Chinese Academy of Sciences, Beijing, China; d Center for Microbial Ecology and Technology, Ghent University, Ghent, Belgium; e Laboratory for Marine Fisheries Science and Food Production Processes, Qingdao National Laboratory for Marine Science and Technology, Qingdao, China; f Key Laboratory of Maricultural Organism Disease Control, Ministry of Agriculture and Rural Affairs, Qingdao, China; g Qingdao Key Laboratory of Mariculture Epidemiology and Biosecurity, Qingdao, China; h Yellow Sea Fisheries Research Institute, Chinese Academy of Fishery Sciences, Qingdao, China; i National Center for Infectious Diseases, Chinese Center for Disease Control and Prevention, Beijing, China; j College of Food Science and Technology, Shanghai Ocean University, Shanghai, China; k School of Biotechnology and Biomolecular Sciences, University of New South Wales (UNSW), Sydney, NSW, Australia; l College of Animal Science and Technology, Yangzhou University, Yangzhou, China; Cleveland Clinic

**Keywords:** *Vibrio parahaemolyticus*, environmental adaptation, genetic exchange, insertion sequence, transmission mode

## Abstract

Vibrio parahaemolyticus is an important foodborne pathogen and has recently gained particular notoriety because it causes acute hepatopancreatic necrosis disease (AHPND) in shrimp, which has caused significant economic loss in the shrimp industry. Here, we report a whole-genome analysis of 233 V. parahaemolyticus strains isolated from humans, diseased shrimp, and environmental samples collected between 2008 and 2017, providing unprecedented insight into the historical spread of AHPND. The results show that V. parahaemolyticus is genetically diverse and can be divided into 84 sequence types (STs). However, genomic analysis of three STs of V. parahaemolyticus identified seven transmission routes in Asia since 1996, which promoted the transfer of an AHPND-associated plasmid. Notably, the insertion sequence (IS*Val1*) from the plasmid subsequently mediated the genetic exchange among V. parahaemolyticus STs and resulted in the deletion of an 11-kb region regulating cell mobility and the production of capsular polysaccharides. Phenotype assays confirmed that this deletion enhanced biofilm formation, providing a novel mechanism for environmental adaptation. We conclude that the transmission mode of AHPND consists of two steps, the transmission of V. parahaemolyticus and the subsequent horizontal transfer of the AHPND-associated plasmid. This plasmid allows IS*Val1* to mediate genetic exchange and improve pathogen fitness in shrimp ponds. Current shrimp farming practices promoted such genetic exchanges, which highlighted a risk of the emergence of new virulent populations, with potentially devastating consequences for both aquaculture and human health. This study addressed the basic questions regarding the transmission mechanism of AHPND and provided novel insights into shrimp and human disease management.

**IMPORTANCE** Global outbreaks of shrimp acute hepatopancreatic necrosis disease (AHPND) caused by V. parahaemolyticus represent an urgent issue for the shrimp industry. This study revealed that the transmission mode of AHPND consists of two steps, the transregional dissemination of V. parahaemolyticus and the horizontal transfer of an AHPND-associated plasmid. Surprisingly, the introduction of the AHPND-associated plasmid also offers a novel mechanism of genetic exchange mediated by insertion sequences, and it improved the fitness of V. parahaemolyticus in a harsh environment. The results presented herein suggest that current shrimp farming practices promote genetic mixture between endemic and oceanic V. parahaemolyticus populations, which introduced the plasmid and accelerated bacterial adaptation by the acquisition of ecologically important functions. This entails a risk of the emergence of new virulent populations both for shrimp and humans. This study improves our understanding of the global dissemination of the AHPND-associated plasmid and highlights the urgent need to improve biosecurity for shrimp farming.

## Abstract (precis)

Global outbreaks of shrimp acute hepatopancreatic necrosis disease (AHPND) caused by V. parahaemolyticus represent an urgent issue for the shrimp industry. This study revealed that the transmission mode of AHPND consists of two steps, the transregional dissemination of V. parahaemolyticus and the horizontal transfer of an AHPND-associated plasmid. Surprisingly, the introduction of the AHPND-associated plasmid also offers a novel mechanism of genetic exchange mediated by insertion sequences, and it improved the fitness of V. parahaemolyticus in a harsh environment. The results presented herein suggest that current shrimp farming practices promote genetic mixture between endemic and oceanic V. parahaemolyticus populations, which introduced the plasmid and accelerated bacterial adaptation by the acquisition of ecologically important functions. This entails a risk of the emergence of new virulent populations both for shrimp and humans. This study improves our understanding of the global dissemination of the AHPND-associated plasmid and highlights the urgent need to improve biosecurity for shrimp farming.

## INTRODUCTION

Vibrio parahaemolyticus is a Gram-negative, halophilic bacterium that is widespread in warm estuarine and marine environments (1). As an important foodborne pathogen, V. parahaemolyticus is becoming the leading cause of acute gastroenteritis due to the increased consumption of raw or undercooked seafood. Recently, V. parahaemolyticus has gained particular notoriety because it causes massive acute hepatopancreatic necrosis disease (AHPND) in shrimp (1, 2). This disease has resulted in economic losses of over $50 billion in global shrimp aquaculture (3). A recent study suggested that V. parahaemolyticus can cause AHPND symptoms in shrimp due to the acquisition of a 70-kb plasmid encoding the binary toxin PirAB^vp^ (4). Evidence of the transfer of *pirAB*-bearing plasmids between different *Vibrio* species has been found (5, 6). The *pirAB* genes and their flanking genes form a mobile genetic element (MGE) called the *pirAB*-Tn*903* composite transposon, or Tn*6264* (6); it consists of six genes and two identical insertion sequences named IS*Val1*.

AHPND was first identified in China and Vietnam in 2010 (7, 8), and it was reported in Malaysia and Thailand in 2011 (9, 10). Afterwards, it was subsequently detected in Mexico and South America (11, 12). The transmission of V. parahaemolyticus was speculated according to this timeline, which has caused long-term disputes in the international shrimp trade between Asia and Mexico. However, our previous genomic study revealed that multiple lineages of V. parahaemolyticus have emerged independently worldwide, with no clear patterns of transmission (2), which is inconsistent with the massive cyclic dysentery epidemics reported in Asia and other countries around the world. To date, very little is known about the origins and spread of V. parahaemolyticus in shrimp farming regions. As farmers often sell diseased shrimp with a high load of the pathogen in the market to reduce economic loss, massive AHPND outbreaks may also pose a threat to public health.

A recent study revealed the existence of binary toxin PirAB^vp^-bearing *Vibrio* spp. isolated from shrimp long before 2010 (12), suggesting the possibility of long-term concealment of AHPND-causing V. parahaemolyticus (*Vp*_AHPND_) strains, without an epidemic outbreak. In our recent study, we found *Vp*_AHPND_ in shrimp most likely introduced from the sediment (13). However, it is still unclear how environmental V. parahaemolyticus strains emerged and adapted to the environment, resulting in shrimp and human disease.

The aim of this study is 2-fold. First, we aimed to understand why multiple lineages of V. parahaemolyticus rather than a single clone emerged in AHPND outbreaks. To this end, a set of V. parahaemolyticus isolates from humans, shrimp, and the environment were selected from the shrimp farming region. Whole-genome analysis was then carried out to reconstruct the spatial and temporal spread of V. parahaemolyticus and to understand whether horizontal plasmid transfer occurred among different V. parahaemolyticus populations. Second, we aimed to determine whether frequent genetic exchanges among V. parahaemolyticus populations were mediated by an insertion sequence from the AHPND-associated plasmid, and we examined its implications for environmental adaptation. To avoid confusion over the terminology used in this study, the AHPND-associated plasmid is referred to as the *pirAB*-positive plasmid.

## RESULTS

### The heterogeneity of *Vp*_AHPND_ strains was possibly a result of horizontal plasmid transfer.

Previous studies have suggested that *Vp*_AHPND_ strains consist of various sequence types (STs) without a pattern of transmission (2), which raises the question of whether many STs were transmitted around the globe and promote the transfer of AHPND-associated plasmids. To this end, a total of 108 V. parahaemolyticus strains were sequenced using the Illumina HiSeq platform (see Data Set S1 in the supplemental material). Together with 125 public genomes (Data Set S2), *in silico* multilocus sequence typing (MLST) showed that the 233 V. parahaemolyticus isolates were classified into 84 STs, of which 20 STs were associated with AHPND outbreaks. ST415 was the most abundant, followed by ST424, ST970, ST2013, ST1166, ST809, and ST150, which constituted 18.75%, 13.54%, 8.3%, 7.29%, 7.29%, 5.21%, and 5.21% of the isolates, respectively.

10.1128/mSystems.00799-19.7 DATA SET S1 General features of strains sequenced in our collection. Download Data Set S1, XLSX file, 0.1 MB. Copyright © 2020 Fu et al. 2020 Fu et al. This content is distributed under the terms of the Creative Commons Attribution 4.0 International license.

10.1128/mSystems.00799-19.8 DATA SET S2 Public genomes used in this study. Download Data Set S2, XLSX file, 0.1 MB. Copyright © 2020 Fu et al. 2020 Fu et al. This content is distributed under the terms of the Creative Commons Attribution 4.0 International license.

**FIG 1:**
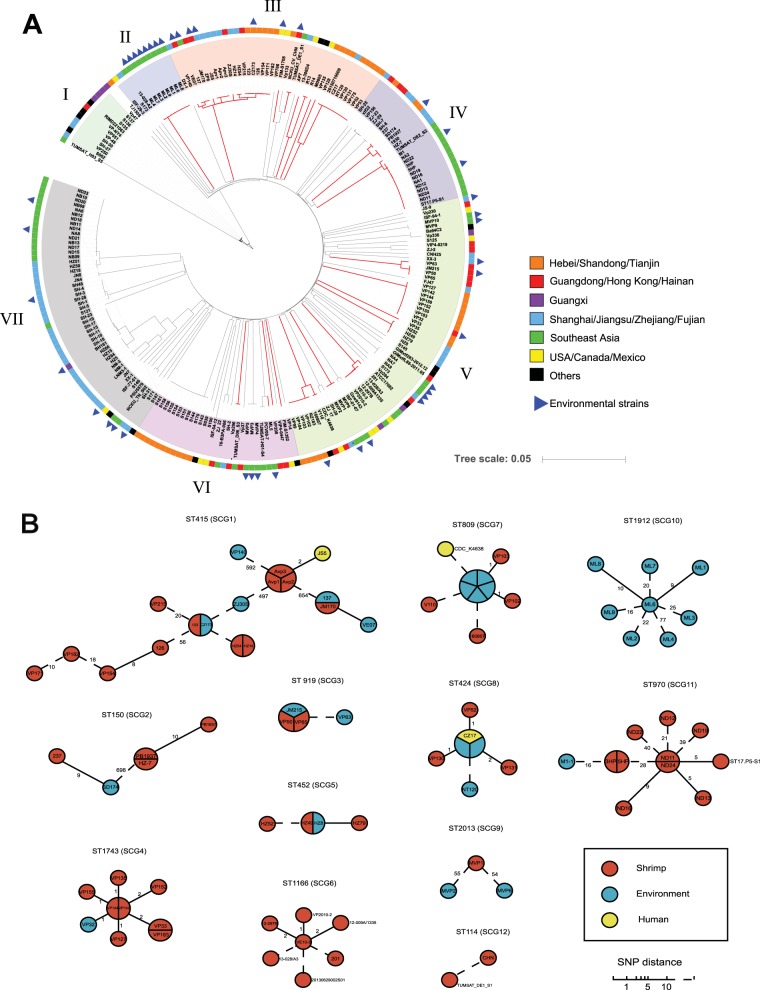
(A) Maximum likelihood (ML) phylogeny of the 233 V. parahaemolyticus genomes isolated from shrimp and humans, showing the seven lineages, I to VII. The strains from different regions are indicated in various colors, as follows: orange, Tianjin, Shandong, and Hebei; red, Guangdong, Hong Kong, and Hainan; purple, Guangxi; yellow, USA/Canada/Mexico; blue, Shanghai, Zhejiang, Jiangsu, and Fujian; green, Southeast Asia; dark, other regions. The branch with *pirAB* is indicated in red. Blue triangle indicates the environmental strains. (B) Minimum spanning trees (MSTrees) of 12 AHPND-related semiclonal groups based on nonrecombined SNPs. Each node indicates a strain. The numbers next to the branches indicate the SNP distance between two nodes.

Subsequent genomic analysis of single-nucleotide polymorphisms (SNPs) in the 233 genomes divided them into seven genetic lineages. V. parahaemolyticus strains associated with AHPND outbreaks (*Vp*_AHPND_) were located on 20 branches in five lineages (Fig. 1A).

By calculating the pairwise SNP distances between all 233 isolates, we defined semiclonal groups (SCG), among which the sequence differences are fewer than 2,000 SNPs, as suggested by Yang et al. (14). Thirty-two SCGs were identified, with each SCG including 2 to 19 isolates. Each SCG only consisted of one ST, except for ST978 and ST1912, which belonged to the same SCG. As we only focused on recently emerged clonal groups in which the recombination was expected to be limited, the pairwise SNP distance was then recalculated for each SCG. Twelve SCGs contained *Vp*_AHPND_ genomes (Fig. 1B). Finally, we identified 40 AHPND-related clones from 12 AHPND-related SCGs in which the pairwise SNP distance was less than 10 (Fig. S1). Interestingly, 10 out of 12 AHPND-related SCGs can be traced back to the environment. Additionally, three SCGs (represented by ST415, ST424, and ST809) included strains from the environment and shrimp and human fecal samples (Fig. 1B), indicating the possible spread of *Vp*_AHPND_ strains from the farm to the table.

10.1128/mSystems.00799-19.2 FIG S1 (A and B) Maximum parsimony tree of 12 semiclonal groups in *Vp*_AHPND_ genomes from China (A) and Southeast Asia (B). The homoplasy index (HI) was 0.0 for the maximum parsimony trees. The numbers above the branches indicate the numbers of SNPs. Download FIG S1, PDF file, 0.2 MB. Copyright © 2020 Fu et al. 2020 Fu et al. This content is distributed under the terms of the Creative Commons Attribution 4.0 International license.

In addition, we found that each SCG came from the same region; no transregional spread was observed in any of the STs except for ST415 (SCG1), ST1166 (SCG6), and ST970 (SCG10). In agreement with this observation, minimum spanning trees (MSTrees) showed a radial pattern of spread for the majority of SCGs, in which one source generated most of the descendants, while secondary spread links were rare, except in SCGs containing the ST415 and ST970 strains (Fig. 1B). Thus, the majority of the *Vp*_AHPND_ strains were genetically diverse instead of originating from one common genotype.

However, previous studies suggested that some *pirAB*-positive plasmids from geographically or genetically distinct strains shared high genetic similarity (2). Therefore, the heterogeneity of *Vp*_AHPND_ genomes might be due to the introduction of the *pirAB*-positive plasmid. However, the occurrence of extensive plasmid transfer events across Asia must meet the following two criteria: (i) the existence of a pandemic clone transmitted from one place to another, and (ii) the observation of plasmid transfer events among various STs in different places. Because both ST415 and ST970 strains came from different countries/regions and showed secondary or more spread links in the MSTrees, we analyze their spatial and temporal transmission patterns in the following section to justify whether they met the above-mentioned criteria.

### Transmission of ST415 V. parahaemolyticus promoted the horizontal plasmid transfer.

**FIG 2:**
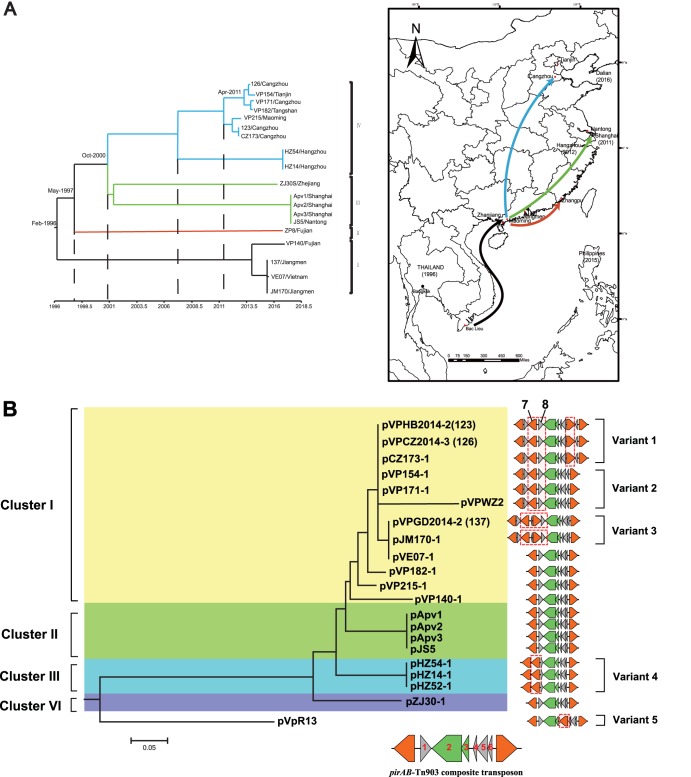
(A) Maximum likelihood (ML) phylogeny of the 19 ST415 genomes studied and their geographic distribution. The tips of the tree are colored to indicate the transmission events. The year of isolation is indicated below. Red points indicate the isolation sites in this study, and black points indicate the isolation sites recorded in the PubMLST database. (B) ML phylogeny of the *pirAB*-positive plasmids from ST415 strains. The variant number of each plasmid is indicated on the right side of the tree. Tn*6264* consisting of six genes (namely, genes 1 to 6) and two identical insertion sequences named IS*Val1* are located under the tree. The insertion of the redundant IS*Val1* is labeled as a dashed line.

The genomic analysis of the 19 ST415 genomes revealed that ST415 strains could be divided into four lineages with strong geographical affinities (Fig. 2A), as follows: lineage I in Vietnam and South China (with isolates collected between 2008 and 2014), lineage II in Fujian Province, China (collected in 2011), lineage III in East China (Zhejiang and Shanghai, collected in 2014), and lineage VI in North China (Hebei and Shandong, 2015 to 2016).

The SNP mutation rate estimated by BEAST yielded a substitution rate of 1.40 × 10^−6^ site^−1^ year^−1^ (95% highest posterior density [HPD], 1.29 × 10^−6^ to 1.51 × 10^−6^) or 2.9 SNPs per genome per year for the ST415 clone. The relaxed clock models and strict clock models yielded nearly identical clock rates. Hence, our results are in line with the strict clock model with a constant population size. The molecular rate is similar to that observed in the V. parahaemolyticus ST36 clone (15). Divergence time analysis of 19 ST415 strains classified them into three stages. BEAST analysis indicated that the most recent common ancestor of ST415 was in February 1996 (95% HPD, May 1995 to October 1997), which is consistent with the time of the first identification of ST415 in Thailand according to the PubMLST database (in 1996). These results suggest that ST415 clones had been established over a long period of time before the AHPND outbreak. By May 1997, ST415 was transmitted to Zhangpu, Fujian Province (lineage II). Afterwards, ST415 spread into East China and diversified (lineage III), as there were a few hundred SNPs on the branch. This stage was estimated to have occurred around October 2000. ST415 subsequently spread to North China by April 2011. Therefore, this ST likely spread as a pandemic clone, resembling the dissemination of the ST3 pandemic in early 1996.

We further extracted and sequenced the plasmids from 19 ST415 genomes. *pirAB*-positive plasmids were identified in all ST415 genomes except for ZP8. Interestingly, sequence analysis showed that the *pirAB*-positive plasmids from 10 ST415 strains, as well as plasmids pVpR14, pHZ52-1, and pVPWZ2, harbored one or two redundant IS*Val1* sequences. Together with the above-mentioned plasmids, the *pirAB*-positive plasmids can be divided into four clusters (Fig. 2B). Overall, the clustering of the plasmids largely reflects the evolutionary relationships of the ST415 chromosomes, except for the plasmids from the Vietnam and Jiangmen (China) strains, which clustered with those obtained from northern China. Genetic analysis showed that redundant IS*Val1* sequences are inserted in different positions in Tn*6264* and that they can be divided into six different variants (Fig. 2B). In addition to two flanking IS*Val1* sequences, Tn*6264* consists of six genes (namely, genes 1 to 6). Variant 1 harbors an additional IS*Val1* (gene 7) and a hypothetical protein (gene 8) inserted between genes 1 and 2 (*pirB*) and an additional IS*Val1* inserted between genes 5 and 6. In contrast to variant 1, variant 2 only has one IS*Val1* inserted between genes 5 and 6. Variant 3 has a second redundant IS*Val1* inserted between genes 7 and 8. For variant 4, gene 1 was replaced by IS*Val1* and by another hypothetical protein (gene 9). Interestingly, pVpR13 (variant 5) also harbors a redundant IS*Val1* inserted next to *pirA* (gene 3). It seems that IS*Val1* has been randomly inserted into different positions in Tn*6264* and that it shaped the genome plasticity of *pirAB*-positive plasmids. In the next section, we further examine the transferability of IS*Val1* from plasmid to chromosome. The variability of Tn*6264* in ST415 strains also suggests that *pirAB*-positive plasmids become genetically diverse during the transmission of ST415 strains.

Likewise, the genetic analysis of AHPND-associated plasmid in *Vp*_AHPND_ strains from Southeast (SE) Asia also suggested that the emergence of ST970 *Vp*_AHPND_ strains facilitated the horizontal transfer of plasmids among different STs in SE Asia, thus promoting the dissemination of AHPND endemically (Text S1 and Fig. S2). Phylogenetic analysis of 88 assembled *pirAB*-positive plasmids revealed extensive plasmid transfer among the common STs found in this study (Fig. S3). This enabled us to reconstruct the historical dissemination of AHPND-associated plasmids in Asia assisted by the transmission of ST970 (transmission route 1 [T1]), ST415 (T2 and T4 to T7), and ST1166 (T3) (Fig. S4).

10.1128/mSystems.00799-19.1 TEXT S1 Phylogenetic analysis of the *pirAB*-positive plasmid. Download Text S1, DOCX file, 0.1 MB. Copyright © 2020 Fu et al. 2020 Fu et al. This content is distributed under the terms of the Creative Commons Attribution 4.0 International license.

10.1128/mSystems.00799-19.3 FIG S2 Maximum likelihood (ML) phylogeny of the *pirAB*-positive plasmid from Southeast Asia. The sequence type of each V. parahaemolyticus strain bearing a *pirAB*-positive plasmid is indicated in parentheses. Possible horizontal plasmid transfer is indicated by a two-way arrow. Download FIG S2, PDF file, 0.3 MB. Copyright © 2020 Fu et al. 2020 Fu et al. This content is distributed under the terms of the Creative Commons Attribution 4.0 International license.

10.1128/mSystems.00799-19.4 FIG S3 Maximum parsimony tree of 88 *pirAB*-positive plasmids from *Vp*_AHPND_. Strain names are indicated in parentheses. Based on the definition of the plasmid clone (PC), the plasmids are divided into six lineages and 25 PCs, indicated in the left. Possible plasmid transfer is indicated in a cycle or a two-way arrow. Black numbers next to branches indicate the number of SNPs contributing to that branch. Download FIG S3, PDF file, 0.2 MB. Copyright © 2020 Fu et al. 2020 Fu et al. This content is distributed under the terms of the Creative Commons Attribution 4.0 International license.

10.1128/mSystems.00799-19.5 FIG S4 Overview of the transmission associated with plasmid transfer in Asia. The arrows are colored to indicate possible transmission events. The different STs of *Vp*_AHPND_ strains bearing *pirAB*-positive plasmids are indicated on the map using the ArcGIS Desktop 10.2 software. The plasmid clone (PC) is indicated in the circle. CHN, China; TH, Thailand; VE, Vietnam; MA, Malaysia. Download FIG S4, PDF file, 0.1 MB. Copyright © 2020 Fu et al. 2020 Fu et al. This content is distributed under the terms of the Creative Commons Attribution 4.0 International license.

### Horizontal plasmid transfer promoted the genetic exchanges of V. parahaemolyticus.

**FIG 3:**
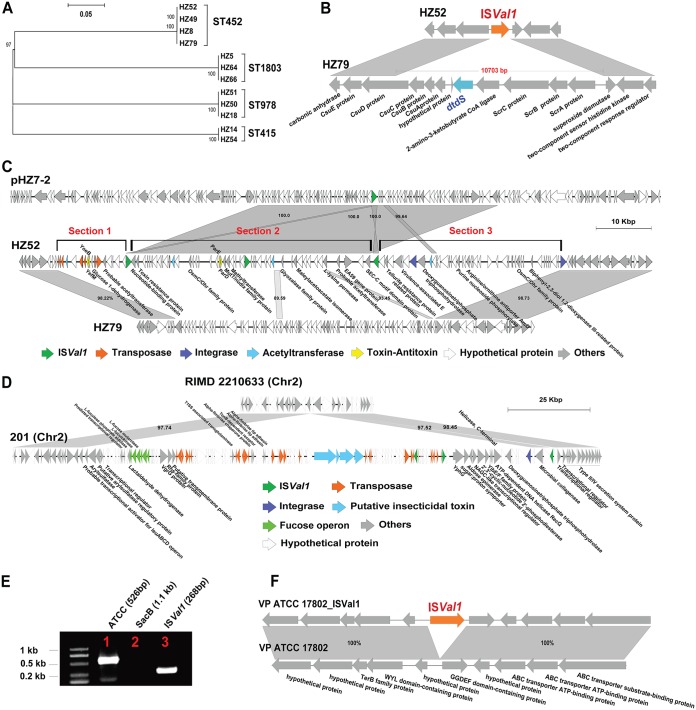
Genetic divergence of V. parahaemolyticus strains from 2014 to 2016 in a shrimp farm of Hangzhou. (A) Maximum likelihood tree of V. parahaemolyticus obtained from a shrimp farm in Hangzhou. (B) Insertion of IS*Val1* into the chromosome and the replacement of a 11-kb genomic island in HZ52. (C) Insertion of a 90-kb genomic island mediated by IS*Val1* in strain HZ52. (D) Insertion of a genomic island mediated by IS*Val1* in strain 201. (E) Insertion of IS*Val1* into the chromosome by *in vitro* experiment. Validation for the insertion of IS*Val1* into the chromosome by PCR after 10 days of laboratory evolution experiments. Lanes 1, 2, and 3 represent PCR products using primers ATCC-F/ATCC-R (V. parahaemolyticus ATCC 17802 chromosomal primers), SacB-F/SacB-R (plasmid-specific primers), and IS*Val1*-268F/IS*Val1*-268R (IS*Val1* specific primers), respectively. (F) The insertion site of IS*Val1* in ATCC 17802 chromosome.

Next, we conducted 3 years of consecutive monitoring in a shrimp farming region to observe the plasmid transfer and its subsequent consequences *in vivo*. In December 2014, strains of ST452, ST1803, and ST978 were isolated from the sediment of shrimp ponds that had not been used for rearing shrimp (Data Set S3). The rearing of postlarval shrimp started in May 2015. No V. parahaemolyticus was detected in the postlarval shrimp before the introduction of shrimp into the pond. Thereafter, we revisited the farm after the suspected AHPND outbreaks occurred (June 2015). Strains HZ14 (ST415), HZ15 (ST1803), and HZ18 (ST978) were identified in the shrimp. These observations suggest that ST1803, ST978, and ST452 are likely to be the endemic STs, while ST415 was introduced to the shrimp farm after 2014. However, during the sampling in June 2016, suspected AHPND outbreaks occurred again. The genotypes in the shrimp pond became genetically diverse. A total of 12 strains were isolated in 2016. MLST subtyped them into ST415, ST452, ST978, ST1803, and an undefined ST with no PCR amplification for the gene *dtdS* (encoding threonine dehydrogenase). ST415 and undefined ST strains both harbored *pirAB*-positive plasmids, which carried a redundant IS*Val1.* Phylogenetic analysis of their chromosomes found that two ST452 strains clustered with two undefined ST strains (Fig. 3A), indicating that this undefined ST might have originated from ST452.

10.1128/mSystems.00799-19.9 DATA SET S3 Genetic features of V. parahaemolyticus isolated from a shrimp farm in Hangzhou. Download Data Set S3, XLSX file, 0.1 MB. Copyright © 2020 Fu et al. 2020 Fu et al. This content is distributed under the terms of the Creative Commons Attribution 4.0 International license.

We also analyzed the plasmid profiles for the sequenced strains in different years. In 2014 and 2015, the strains from ST452, ST1803, and ST978 all harbored a 106-kb *pirAB*-negative plasmid (plasmid 1, Data Set S3). However, since 2015, when the HZ14 strain (harboring a 69-kb *pirAB*-positive plasmid [plasmid 2] and a 76-kb *pirAB*-negative plasmid [plasmid 3]) was identified, the plasmid profiles of ST452 have changed. Afterward, plasmids 2 and 3 were transferred from ST415 to ST452 with a *dtdS* deletion, while normal ST452 only acquired plasmid 3. The ST1803 and ST978 strains still only harbored plasmid 1 (Data Set S3).

Remarkably, the genomic analysis of two undefined ST strains showed that the insertion of IS*Val1* resulted in the loss of an 11.3-kb DNA fragment enclosing the *dtdS* locus (Fig. 3B). In addition, the insertion of IS*Val1* was identified in various locations of the chromosomes of strains HZ52 and HZ49 as well as in their plasmids. Further analysis showed that a total of 24 IS*Val1* insertions were found throughout the genome of strain HZ52, including a replacement event, five deletion events, and two insertion events (Table 1).

**TABLE 1:** Insertion sites of IS*Val1* and subsequent genetic changes in HZ52 genome

Location^*a*^	IS*Val1* no.	Genome positions in V. parahaemolyticus HZ52		Type(s) of genetic changes (size [kb])^*b*^
		Start	Stop	
Chr 1	1	11566	12486	I
	2	160107	159187	GI (∼16)
	3	658972	659892	I
	4	781511	780591	I
	5	1034454	1035374	I
	6	1040909	1041829	I
	7	1579626	1580546	GI (∼90) and SD (∼54)
	8	1601166	1602086	
	9	1625354	1624434	
	10	1898558	1897638	I
	11	3036111	3035191	I
	12	3305121	3306041	SD (∼0.8)
Chr 2	13	325274	326194	SD (∼14)
	14	664878	665798	SD (∼0.5)
	15	712965	713885	I
	16	795073	794153	SD (∼10) *dtdS* lacking
	17	1023249	1022329	I
	18	1105782	1106702	I
	19	1150074	1149154	I
	20	1156740	1155820	I
	21	1288735	1287815	I
	22	1569276	1570196	I
	23	1640600	1639680	SD (∼2.2)
pHZ52-3	24	22284	21364	I

a Chr, chromosome.

b I, insertion of IS*Val1* only; GI, genomic island obtained in V. parahaemolyticus HZ52 mediated by IS*Val1*; SD, sequence deletion of V. parahaemolyticus HZ52 mediated by IS*Val1*.

In strain HZ52, a 54-kb segment encoding the YefM-YoeB toxin-antitoxin system, acetyltransferase, and proteins encoded a few genes associated with metabolism was replaced by a 90-kb genomic island (Fig. 3C). This genomic island was flanked by the IS*Sod13* transposase and integron integrase IntI4 with three IS*Val1* copies inserted. Interestingly, this genomic island can be divided into three sections. The first section was found in the chromosome of strain HZ-7 (isolated from another shrimp farm in Hangzhou), while the second section separated by IS*Val1* was located in a plasmid from HZ-7. However, the origin of the third section remains unclear. Thus, it is reasonable to speculate that IS*Val1* might mediate the excision of the first section, be incorporated with the second section, and transferred as a whole into HZ52. However, a gene-by-gene analysis showed that this genomic island is functionally similar to the replaced segment, as the two harbored a similar set of genes. The inserted genomic island carried an additional toxin-antitoxin system.

The five deletion events in strain HZ52 resulted in the loss of genes encoding a TonB-dependent receptor, *dtdS* and its surrounding region, acetyltransferase/long-chain fatty acid transport protein/ADP-ribose pyrophosphatase, endonuclease I, and Flp pilus assembly. An insertion event introduced genes encoding a type I restriction-modification system. In addition, plasmid 3 in strain HZ49 harbored an additional 12-kb drug-resistant island (DRI) flanked by two IS*Val1* copies. Five antibiotic resistance genes were identified in the DRI, *aadA16*, *ARR-3*, *sul1*, *tet*(B), and *dfrA27*. This DRI has also been found in the plasmid pVPSD2016-2 from strain 237 but without IS*Val1*, indicating that IS*Val1* might facilitate the formation of composite transposons and be transferred between plasmids.

Likewise, we also found a 127-kb genomic island in strain 201 and another ST1166 *Vp*_AHPND_ strain, which was also possibly associated with the introduction of the *pirAB*-positive plasmid (Fig. 3D). This genomic island harbored three IS*Val1* copies that formed another composite transposon.

To confirm whether IS*Val1* can move between plasmids and chromosomes, an *in vitro* evolutionary experiment was conducted. A plasmid containing two IS*Val1* copies was constructed and cocultured with V. parahaemolyticus strain ATCC 17802 for 10 days. At the end of the experiment, the disappearance of the plasmid occurred when *sacB* (located on the plasmid) was not detected through PCR (Table 2). At day 10, a total of 58 colonies were randomly selected for PCR detection of IS*Val1* and *sacB.* Four out of 58 colonies were IS*Val1* positive and *sacB* negative, indicating that the insertion rate of IS*Val1* is 6.9%. One of the colonies was selected for genome sequencing. The results confirmed that IS*Val1* was transferred from the constructed plasmid (Fig. S5) to chromosome I (GenBank accession no. CP014046.2) of V. parahaemolyticus (ATCC 17802) at positions 614539 to 614547 (Fig. 3E and F).

**TABLE 2:** PCR primers used in this study

Primer	Sequence (5′–3′)	Reference
cpsA-F	GAGAGCGGCAACCTATATCG	Zhang et al. (40)
cpsA-R	GCGGTCAAACAAAGGGTAAAC	Zhang et al. (40)
IS*Val1*_268F	GCTTAAATACGGAGTCTAG	This study
IS*Val1*_268R	ACGCCCATCAATCGTCGT	This study
ATCC_526F	GAAGGATGTCAGAGAAACACTGTAT	This study
ATCC_526R	AGAATACTTTGTTACTGTCGAGGC	This study
SacB-1120F	ATACTTTGGCGTCACCCCTTAC	This study
SacB-1120R	GCGGTTTCATCACTTTTTTCAG	This study
IS*Val*-3.2kF	CTAGCTCAGTCCTAGGTACAGTGCTACTTGGCAGCGAAGCTATATTGTGAA	This study
IS*Val*-3.2kR	GGATACATATTTGAATGCTCGAGCCGTGGTAGAACTAGGCAAGGCTCATA	This study
pYC1000-4.4kF	TATGAGCCTTGCCTAGTTCTACCACGGCTCGAGCATTCAAATATGTATCC	This study
pYC1000-4.4kR	TTCACAATATAGCTTCGCTGCCAAGTAGCACTGTACCTAGGACTGAGCTAG	This study

10.1128/mSystems.00799-19.6 FIG S5 Diagram of the plasmid pYC1000-IS*Val1* that carries two copies of IS*Val1.* Download FIG S5, PDF file, 0.1 MB. Copyright © 2020 Fu et al. 2020 Fu et al. This content is distributed under the terms of the Creative Commons Attribution 4.0 International license.

BLASTn results show that insertion of IS*Val1* occurred in the chromosomes of 50 *Vp*_AHPND_ and 5 AHPND-associated *Vibrio* sp. strains, with the number of IS*Val1* ranging from 1 to 25 (Data Set S4), but this did not occur in any non-*Vp*_AHPND_ strains. These observations indicate that the insertion of IS*Va11* into the chromosome was caused by the introduction of a *pirAB*-positive plasmid, which provided an underlying mechanism for the transfer of MGEs and virulence genes.

10.1128/mSystems.00799-19.10 DATA SET S4 Insertion of IS*Val1* into the chromosome of V. parahaemolyticus and other *Vibrio* sp. genomes. Download Data Set S4, XLSX file, 0.1 MB. Copyright © 2020 Fu et al. 2020 Fu et al. This content is distributed under the terms of the Creative Commons Attribution 4.0 International license.

### Phenotypic difference between strains HZ52 and HZ79 reveals the role of genetic exchange in environmental adaptation.

**FIG 4:**
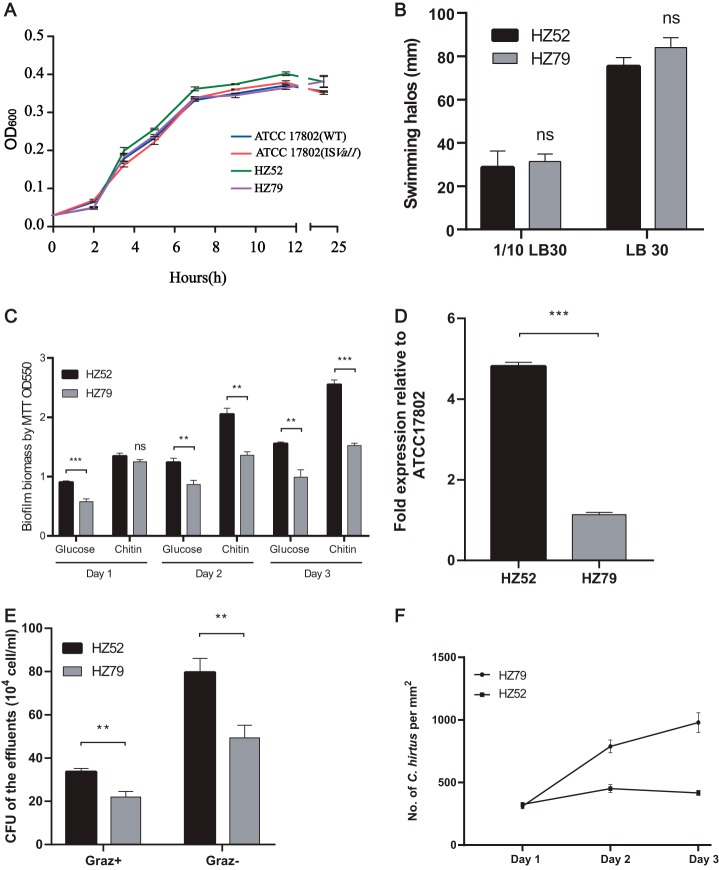
Phenotype assays of strains HZ52 and HZ79. (A) The growth curve of strains HZ52 and HZ79. (B) Swarming mobility assay. (C) Biofilms formed on chitin surfaces or glucose in 24-well plates. (D) Detection of the expression of *cpsA* by quantitative PCR (qPCR). (E) Biofilms formed on chitin surfaces in a flowthrough system (E). Experiments were performed in flow cells for 3 days in the presence (Graz+) and absence (Graz−) of *Coleps hirtus*. The CFU of V. parahaemolyticus biofilm effluents grown on chitin flakes were determined. (F) Effect of biofilms on the survival of *Coleps hirtus* in a flowthrough system. *Coleps hirtus* was quantified by microscopy daily. *, **, and *** indicate significant differences at *P* values of <0.05, <0.01, and <0.001, respectively, while ns indicates no significant difference. All values are expressed as the mean ± standard deviation (SD).

Next, we tested the phenotypic difference between strains HZ52 and HZ79 to determine whether the insertion of IS*Val1* contributed to environmental adaptation. The growth assay showed that there was no significant difference between strains HZ52 and HZ79 in terms of growth rate (Fig. 4A). Likewise, strain ATCC 17802 with the insertion of IS*Val1* also had a growth rate similar to that of its wild type. The growth rates between HZ52 and ATCC 17802 were also not significantly different. In addition, strains HZ52 and HZ79 exhibited similar swimming ability (*P* > 0.05, Fig. 4B), indicating that the loss of the *scr* and *csu* operons was not determinative for mobility. Biofilm formation assays showed that the biomass of biofilms for strain HZ52 was significantly greater than that for strain HZ79 formed on chitin flakes and glucose on days 2 and 3 (*P* < 0.01, Fig. 4C). In addition, strain HZ52 exhibited 4.2-fold higher *cpsA* expression relative to HZ79 (*P* < 0.001), indicating higher capsular polysaccharide (CPS) production (Fig. 4D).

To determine whether HZ52 also exhibited a higher biofilm formation ability under continuous-flow and -grazing conditions, V. parahaemolyticus strains and ciliate Coleps hirtus were coincubated in flow cells with chitin. The grazing effects were determined with the quantification of V. parahaemolyticus CFU and the numbers of *C. hirtus* in the biofilm effluent. The results suggest that with or without grazing pressure, effluents from strain HZ52 both contained approximately 1.5-fold more V. parahaemolyticus cells than did the effluents from strain HZ79 (*P* < 0.01, Fig. 4E). Direct microscopic enumeration also revealed that the number of *C. hirtus* cells increased in the flow cell system inoculated with strain HZ79, relative to the one inoculated with strain HZ52 (Fig. 4F) on day 3, although the differences between the two groups were not significant.

## DISCUSSION

### Horizontal plasmid transfer promoted the genetic exchange and environmental adaptation for *Vp*_AHPND_ strains.

In this study, we provide the first global analysis of the transmission pattern of *Vp*_AHPND_ strains by genomic epidemiology. The results herein suggest that the transmission of a few genotypes of *Vp*_AHPND_ strains is an important driving force promoting the transfer of *pirAB*-positive plasmids and AHPND. The temporal and spatial analysis of ST415 strains suggests that ST415 is possibly another pandemic clone that was transmitted simultaneously with the pandemic ST3, which has been transmitted along the coastline of China in sequential order from 1996 to 2010 (16). However, due to insufficient sequenced strains from the Americas, it is still not clear how AHPND spread from Asia to South America. Another limitation of this study is that horizontal plasmid transfer events were only epidemiologically observed at one site. Nevertheless, given the observations by Dong et al. (17), plasmid transfer among different *Vibrio* spp. is likely. Extensive plasmid transfer events seemed to occur during the dissemination of ST415, ST1166, and ST970, which greatly promoted the spread of AHPND in Asia and accelerated genetic mixing. With the exception of the above-mentioned three STs, the STs remained endemically present in each region and became *Vp*_AHPND_ strains after receiving the plasmid.

**FIG 5:**
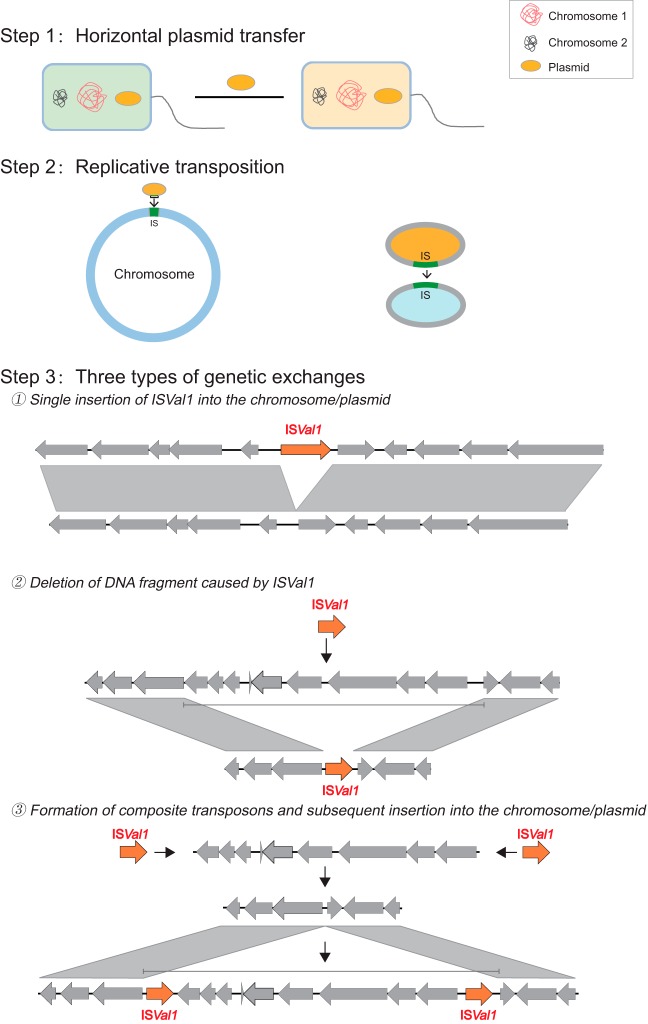
Schematic diagrams of the details of how IS*Val1* mediates the genetic changes in the chromosome. Three types of genetic changes were identified: 1. Single insertion of IS*Val1* into the chromosome/plasmid; 2. Deletion of DNA fragment caused by IS*Val1*; 3. Formation of composite transposons and subsequent insertion into the chromosome/plasmid.

Unexpectedly, the introduction of the *pirAB*-positive plasmid also promoted genetic mixing within V. parahaemolyticus populations caused by several insertion and deletion events. Observation on a shrimp farm showed that *pirAB*-negative (avirulent) strains were present in the environment until the introduction of ST415. Afterwards, ST452 strains acquired both *pirAB*-positive and *pirAB*-negative plasmids from ST415 strains present in the local environment. Thereafter, IS*Val1* excised from the *pirAB*-positive plasmid, resulting in three types of genetic changes for ST452 strains (Fig. 5), including an 11-kb deletion in the region surrounding *dtdS*. These observations suggest that the transmission of V. parahaemolyticus and subsequent horizontal plasmid transfer might confer upon IS*Val1* the ability to mediate the transfer of MGEs. It seems that IS*Val1* excised from the plasmid to the chromosome by replicative transposition instead of cut-and-paste transposition as in the *pirAB*-positive plasmid from ST415 and ST452 harbored the redundant IS*Val1.* Along with plasmid excision, transposons are faced with the task of locating new insertion sites in the genome to integrate within. Some transposon elements are very strict in the sequence feature, such as the Tc1/mariner elements, which always integrate into a TA dinucleotide (18). However, the insertion sites for IS*Val1* are more flexible and vary in nucleotide composition, and no integration site preference was observed (Data Set S4). This feature enables IS*Val1* to be a powerful gene vehicle and to insert into both the plasmid and the chromosome.

Among these insertion events, the most interesting one is the deletion of the 11-kb region including the *dtdS* gene, *scr* operon, and *csu* operon which is mostly associated with cell swimming ability. Therefore, HZ52 might have a defective swimming function. However, Boles and McCarter suggested that mutations in the *scrABC* genes greatly reduce but do not eliminate lateral flagellin production; conversely, the loss of the *scr* operon enhanced the production of CPS (19). Therefore, these genes are not absolutely required for cell mobility. In addition, because lateral flagellar production is a costly process that is not beneficial for the cells that are already on the surface, the deletion of the 11-kb segment might control lateral gene expression and enhance CPS production. To test this idea, the expression of a CPS synthase gene (*cpsA*) was monitored in HZ52 and HZ79, and HZ52 showed significant higher *cpsA* expression (*P* < 0.01). Further validation experiments supported the above-mentioned hypothesis. Compared with HZ79, strain HZ52 showed slightly lower cell mobility but significant higher biofilm formation ability under both static and continuous-flow conditions. Thus, strain HZ52 might have better survival in the shrimp pond. Therefore, genetic exchanges mediated by IS*Val1* promoted the insertion and deletion of the genomic island, which might enhance the environmental adaptation of endemic V. parahaemolyticus populations and turn avirulent strains into virulent strains that cause disease in both shrimp and humans.

### Transmission mode of AHPND and its implications for shrimp and human disease management.

**FIG 6:**
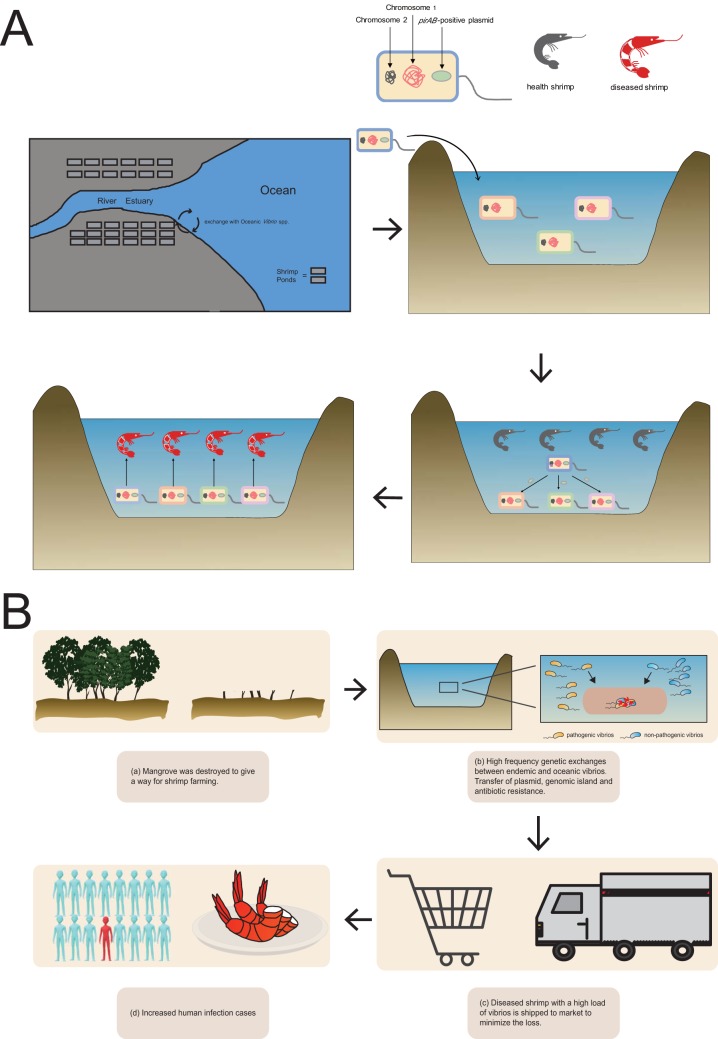
(A and B) Schematic diagrams of the transmission mode of acute hepatopancreatic necrosis disease (AHPND) (A) and how current farming practice promotes the emergent spread of V. parahaemolyticus (B). (A) The transition from non-*Vp*_AHPND_ to *Vp*_AHPND_ is caused by recent transmission of *Vp*_AHPND_ and subsequent horizontal plasmid transfer. (B) a, mangrove was destroyed to provide a way for shrimp farming; b, high-frequency genetic exchanges between endemic and oceanic vibrios, resulting in the transfer of plasmid, genomic island, and antibiotic resistance; c, diseased shrimp with a high load of vibrios is shipped to market to minimize the loss; and d, increased human infection cases caused by the consumption of diseased shrimp.

Our results also suggest that the transmission mode accounting for the occurrence of AHPND has great impacts on shrimp and human disease management (Fig. 6A). In this mode, the endemic V. parahaemolyticus population is *pirAB* free. However, as the majority of shrimp ponds are located in estuary regions with the frequent exchange of oceanic *Vibrio* spp., *pirAB*-positive *Vibrio* spp. might have recently entered the shrimp ponds and thus be responsible for AHPND outbreaks or may have been carried by postlarval shrimp to other regions (20).

This mode provides significant implications for shrimp and human disease management. Epidemiological investigation showed that a large quantity of mangroves were destroyed to make shrimp ponds prior to 2010 in SE Asia and South China, which greatly increased the exposure of shrimp to opportunistic pathogens from the ocean (Fig. 6B). Such farming practices in the coastal region not only disrupted the ecological systems but also increased genetic mixing between pathogenic and nonpathogenic *Vibrio* spp. This aquaculture mode created an environment in which the opportunities of contact between the populations increased, thus maximizing the probability of genetic material transfer and recombination, as observed in Vibrio vulniﬁcus (21). Genetic exchange (via plasmids and genomic islands) at this shrimp farm also presents a good example of how the transfer of antibiotic resistance has taken place between plasmids. He et al. reported that multidrug-resistant V. parahaemolyticus strains were frequently identified in shrimp from Jiangsu Province, China, and suggested that MGE is the main vehicle for the transfer of resistance (22). This study also highlights that the formation of composite transposons mediated by IS*Val1* provides a novel mechanism for antibiotic resistance transfer.

From a One Health perspective, current shrimp farming practices resulting in widespread *Vp*_AHPND_ strains also pose an emergent threat to public health. Current shrimp farming practices have promoted frequent exchange with oceanic vibrios, which could entail a risk of the emergence of virulent populations, with potentially devastating consequences for both aquaculture and human health. By incorporating shrimp, environmental strains, and clinical strains, three farm-to-table spread events were identified. This study also confirmed the transmission of *Vp*_AHPND_ strains from the environment to the farm and to the table genomically. A more comprehensive spread network should be investigated for other SCGs. To minimize loss, diseased shrimp with a high load of vibrios are often shipped to market, resulting in a possible increase in human infections. Thus, improving biosecurity management may be the key to minimizing the progression of AHPND and reducing subsequent human infection risks. The transformation from traditional pond farming to indoor recirculating aquaculture systems would be a promising direction for effective disease control and prevention, as this new aquaculture mode promotes rigorous biosecurity management, which could efficiently prevent the entrance of external pathogens.

The results presented herein suggest that the transmission of ST415 *Vp*_AHPND_ strains, as well as of a few other STs, promoted horizontal plasmid transfer and turned endemic V. parahaemolyticus into virulent *Vp*_AHPND_. This study identified a novel genetic exchange mechanism mediated by IS*Val1*. Current shrimp farming practices have promoted frequent exchange with oceanic vibrios, which could entail a risk of the emergence of virulent populations with potentially devastating consequences for both aquaculture and human health. This study addressed the basic question regarding the origins and evolutionary history of *Vp*_AHPND_, with significant implications for shrimp and human disease management, and highlighted the urgent need to improve the biosecurity of shrimp from the farm to table.

## MATERIALS AND METHODS

### Bacterial isolates and total DNA extraction.

The V. parahaemolyticus isolates analyzed in this study are listed in Data Set S1. Total DNA was extracted from the overnight culture using the Wizard genomic DNA kit (Promega).

### Sample collection and bacterial isolation and identification from a shrimp farm in Hangzhou.

In December 2014, May 2015, June 2015, and June 2016, samples of sediment, postlarval shrimp, and diseased shrimp were collected from a shrimp farm in Hangzhou, China. Hepatopancreases from shrimp and sediments were aseptically disaggregated in 100 ml alkaline peptone water (peptone, 10 g per liter; sodium chloride, 10 g per liter) and streaked on thiosulfate-citrate-bile salts-sucrose (TCBS) plates, which were incubated at 28°C for 12 h.

### MLST.

*In silico* MLST typing of a publicly available V. parahaemolyticus genome was performed using the MLST 2.0 server from the Center for Genomic Epidemiology (23).

### Whole-genome sequencing, *de novo* assembly, and annotation.

High-throughput genome sequencing was carried out on Illumina platforms or a PacBio RS II platform (Novogene, China). The FASTQ reads were quality trimmed with Trimmomatic (v0.36) (24). The draft genome was assembled *de novo* with SPAdes version 3.0 (25). Antimicrobial resistance genes were identified with ResFinder (26). RAST was used to annotate the sequences of each genome determined with next-generation sequencing (27).

### Identification of SNPs, phylogenetic analyses, and definition of clonal group.

The core genome of V. parahaemolyticus defined by Gonzalez-Escalona et al. (28) was used as the reference genome to call the SNPs for V. parahaemolyticus (29). SNPs located in the recombination regions were removed by ClonalFrameML (30). Taking 2,000 as the threshold of pairwise SNP distance between strains (14), we defined semiclonal groups (SCGs) in our analyzed data set. Meanwhile, we recalculated the pairwise SNP distance between isolates of each SCG to identify clones which were defined as the genomes with fewer than 10 SNPs differences (14).

RAxML version 7.8.6 was used with the generalized time-reversible model and a gamma distribution to model site-specific rate variation (the GTR+⌈ substitution model; GTRGAMMA in RAxML) (31). SNPs were recalled for each SCG to gain a higher resolution. ClonalFrameML was used to identify the recombination regions for each SCG. The nonrecombined SNPs were used in GrapeTree (32) to construct the minimum spanning trees. Maximum parsimony algorithms were used for SCG in PAUP 4.0 to precisely identify the SNPs that differed among them (33).

### Temporal analysis.

Bayesian Evolutionary Analysis by Sampling Trees (BEAST) version 1.8.4 was used to date the important nodes (34). The concatenated nonrecombinant chromosomal SNP alignments of ST415 and ST970 strains were subjected to multiple BEAST analyses with both a strict molecular clock and a relaxed clock, in combination with either constant-size or Bayesian skyline population-size models, to identify the best-fit model, respectively. A marginal likelihood estimation was carried out to obtain path sampling (PS)/stepping-stone sampling (SS) values for each run that had converged in order to compare the different combinations of clock and tree models (35).

### Experimental evolution of V. parahaemolyticus and detection of insertion event.

To confirm the transferability of IS*Val1* from plasmid to chromosome, a coculture experiment of V. parahaemolyticus with IS*Val1-*containing plasmids was conducted. To prevent the nonspecific amplification of similar insertion sequences in V. parahaemolyticus ATCC 17802, the specific primer pair IS*Val1*_268F/IS*Val1*_268R (Table 2) was designed for IS*Val1* according to the comparison of the IS*Val1* sequence and ATCC 17802 genome sequence. Two consecutive IS*Val1* sequences were amplified by PCR from the *pirAB*-positive plasmid pHZ52-1 and cloned into the SacB-containing plasmid pYC1000-eforRED (36) using Gibson cloning to yield pYC1000-IS*Val1*. The primers used are shown in Table 2.

Experimental evolution was conducted in a 12-well plate with 2 ml LB30 (LB with 3% NaCl) and 20 μg/ml chloramphenicol per well. Every 24 h, 0.02 ml bacterial culture was transferred to a new 12-well plate and incubated at 15°C and 30°C, respectively, under nine parallel experiments that lasted for 10 days. A 20% sucrose-containing LB agar plate was used to eliminate the plasmid. The loss of the plasmid and insertion of IS*Val1* were detected in sucrose-resistant and chloramphenicol-sensitive strains by PCR using the primer pairs ATCC-526F/ATCC-526R (an ATCC 17802-specific sequence primer pair to verify that the strain is not contaminated), SacB-1120F/SacB-1120R (SacB gene-specific primers to confirm that the plasmid was eliminated), and IS*Val1*_268F/IS*Val1*_268R (an IS*Val1*-specific primer pair to identify IS*Val1* insertion into the genome), which are confirmed by DNA sequencing.

### Bacterial growth assay.

Triplicates of overnight cultures of V. parahaemolyticus strains were normalized to an optical density at 600 nm (OD_600_) of 0.1 in TSB30 broth (TSB plus 3% NaCl) and grown at 30°C in TSB30. Growth was measured as the OD_600_. The assay was repeated three times independently, with similar results.

### Swimming motility assay.

The swimming motility assay was performed on soft LB30 (LB plates containing 0.3% agar) or 1/10 LB30, as described by Yang and Defoirdt (37). V. parahaemolyticus strains were grown overnight in TSB30 broth, and 5-μl aliquots (OD_600_, 1.0) were spotted in the center of the soft agar plates. Plates were incubated for 24 h, after which the diameters of the motility halos were measured. All assays were repeated at three times independently. The values are expressed as the mean ± standard deviation (SD).

### Biofilm formation assay.

To compare biofilm formation abilities on abiotic and chitinous surfaces between HZ52 and HZ79, batch experiments were performed in 24-well culture plates. V. parahaemolyticus overnight cultures were incubated at a final concentration of 10^6^ cells ml^−1^ in 1 ml of marine minimal medium (MMM) (38). Chitin flakes (2% [wt/vol]; Sigma-Aldrich, St. Louis, MO, USA) or 1% (wt/vol) glucose was supplemented as a source of carbon. The microtiter plates were incubated at room temperature with shaking at 60 rpm for 3 days. V. parahaemolyticus biofilm biomass was determined by 3-(4,5-dimethyl-2-thiazolyl)-2,5-diphenyl-2H-tetrazolium bromide (MTT) staining, as described by Sun et al. (39). The experiments were repeated three times. The values are expressed as the mean ± SD.

### RNA isolation and quantitative real-time PCR.

The VPA1403–1412 (*cpsABCDEFGHIJ*) operon is responsible for exopolysaccharide production in V. parahaemolyticus, which is controlled by *scrABC*. To analyze the gene expression of *cpsA* in HZ52 relative to that of the wild-type strain HZ79, we performed relative quantitative reverse transcription-PCR (qRT-PCR) for *cpsA* using methods described by Zhang et al. (40). Briefly, overnight cultures of V. parahaemolyticus strains ATCC 17082, HZ52, and HZ79 were subjected to RNA extraction using the TRIzol reagent (Invitrogen, USA). Total bacterial RNA (1 μg) was reverse transcribed using qScript cDNA SuperMix (Quanta Biosciences). To measure gene expression, 50 ng of cDNA template from each strain was amplified by real-time qRT-PCR (Applied Biosystems 7900 HT) with Power SYBR green master mix. The qRT-PCR assay was performed on three technical and three biological replicates for each sample. The expression levels of each gene were normalized using an endogenous control gene (DNA gyrase subunit B [*gyrB*]) to correct for sampling errors. Fold changes in the levels of gene expression relative to ATCC 17082 were measured using the Pfaffl equation (41). The values are expressed as the mean ± SD.

### Flow cell and grazing assay.

*C. hirtus* used for the grazing assay was isolated from a shrimp pond and routinely grown on heat-killed Vibrio harveyi strain HW0009 (final concentration, 10^6^ cells ml^−1^). V. parahaemolyticus HZ52 and HZ79 were incubated with or without *C*. *hirtus* in a continuous flow cell system, as described by Sun et al. (39). Biofilms of strains HZ52 and HZ79 were grown at room temperature in three-channel (1 by 4 by 40 mm) flow cell tubings, which were attached to a peristaltic pump and continuously fed for 3 days with sterilized marine minimal medium (MMM). One milliliter of MMM containing 10^6^ cells ml^−1^ of V. parahaemolyticus with or without 10^4^ cells ml^−1^ of *C*. *hirtus* was injected into the cell and allowed to settle for 2 h, after which the medium flow was resumed at a rate of 0.3 ml/min. Chitin flakes were glued to the bottoms of the flow cells using glass silicon. *C*. *hirtus* was enumerated daily by inverted microscopy, and the effluents were collected and plated onto TCBS agar to determine the V. parahaemolyticus CFU at day 3. The experiments were repeated three times independently. The values are expressed as the mean ± SD.

### Statistical analyses.

Data analysis was carried out using the SPSS statistical software (version 18). Bacterial growth data were analyzed by one-way analysis of variance (ANOVA) with a Bonferroni correction. Unless stated otherwise, all other data were compared with independent-samples *t* tests. A *P* value of <0.05 was considered to indicate statistical significance.
