# Supplementary figures and images for: Horizontal Plasmid Transfer Promotes the Dissemination of Asian Acute Hepatopancreatic Necrosis Disease and Provides a Novel Mechanism for Genetic Exchange and Environmental Adaptation
Source: mSystems. 2020 Mar 17;5(2):e00799-19. doi: 10.1128/mSystems.00799-19 (PMC7380584; doi:10.1128/mSystems.00799-19)

A

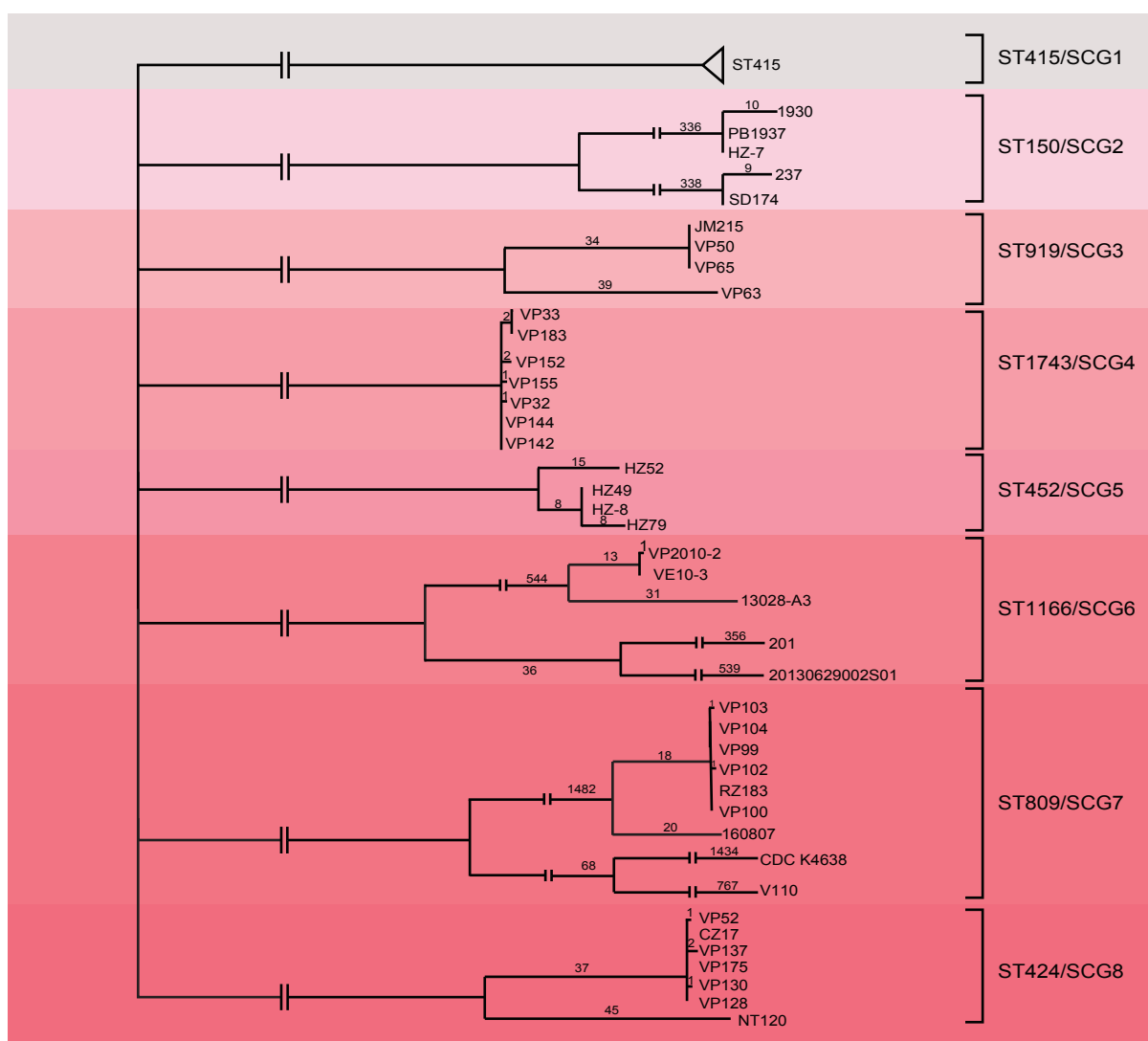

B

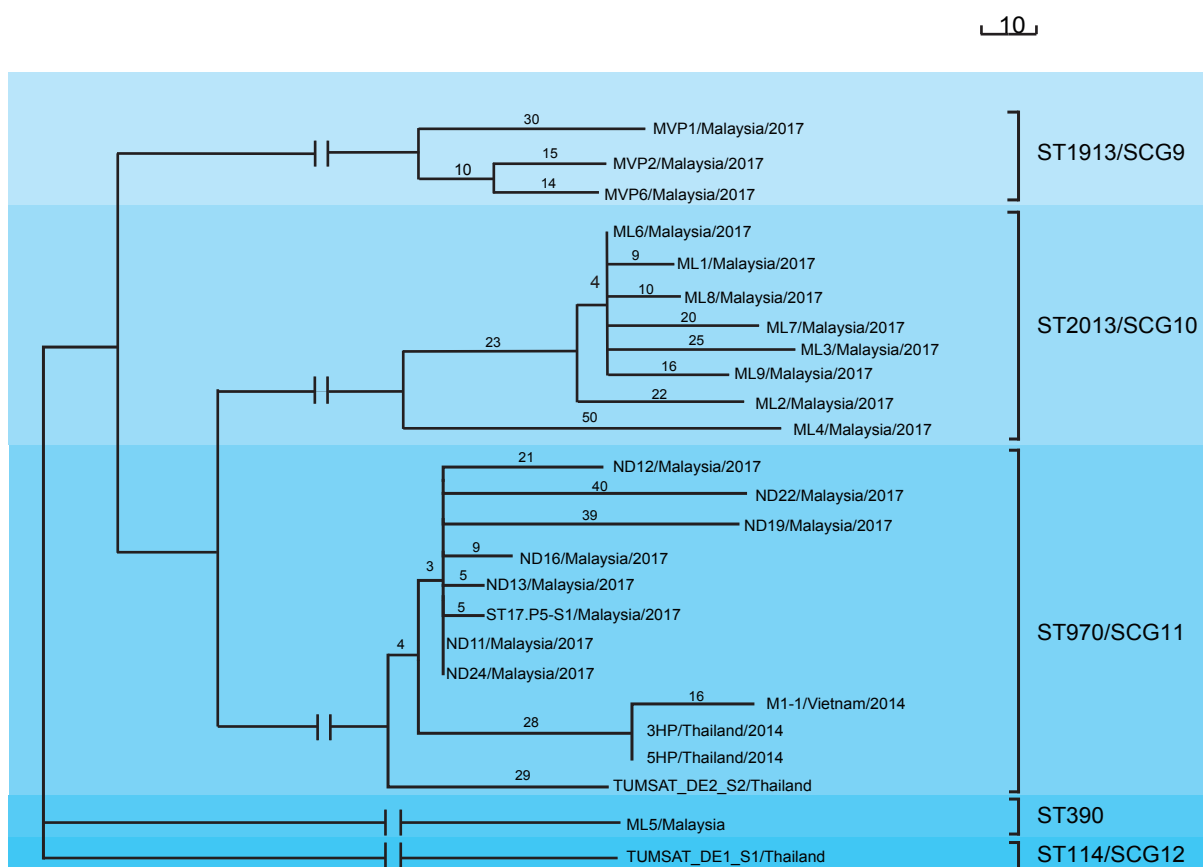

Supplement: FIG S1 [file mSystems.00799-19-sf001.pdf]

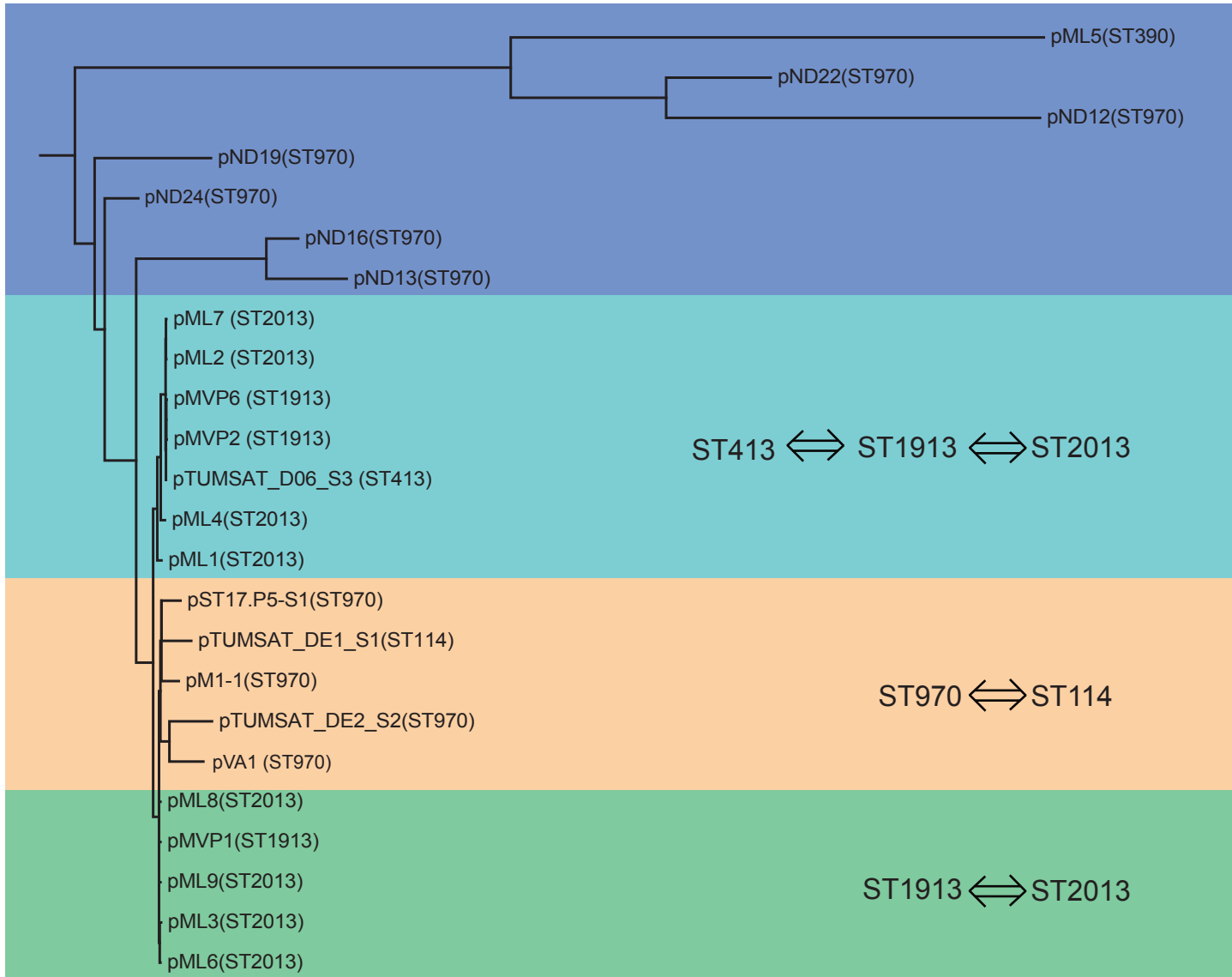

0.1

Supplement: FIG S2 [file mSystems.00799-19-sf002.pdf]

A

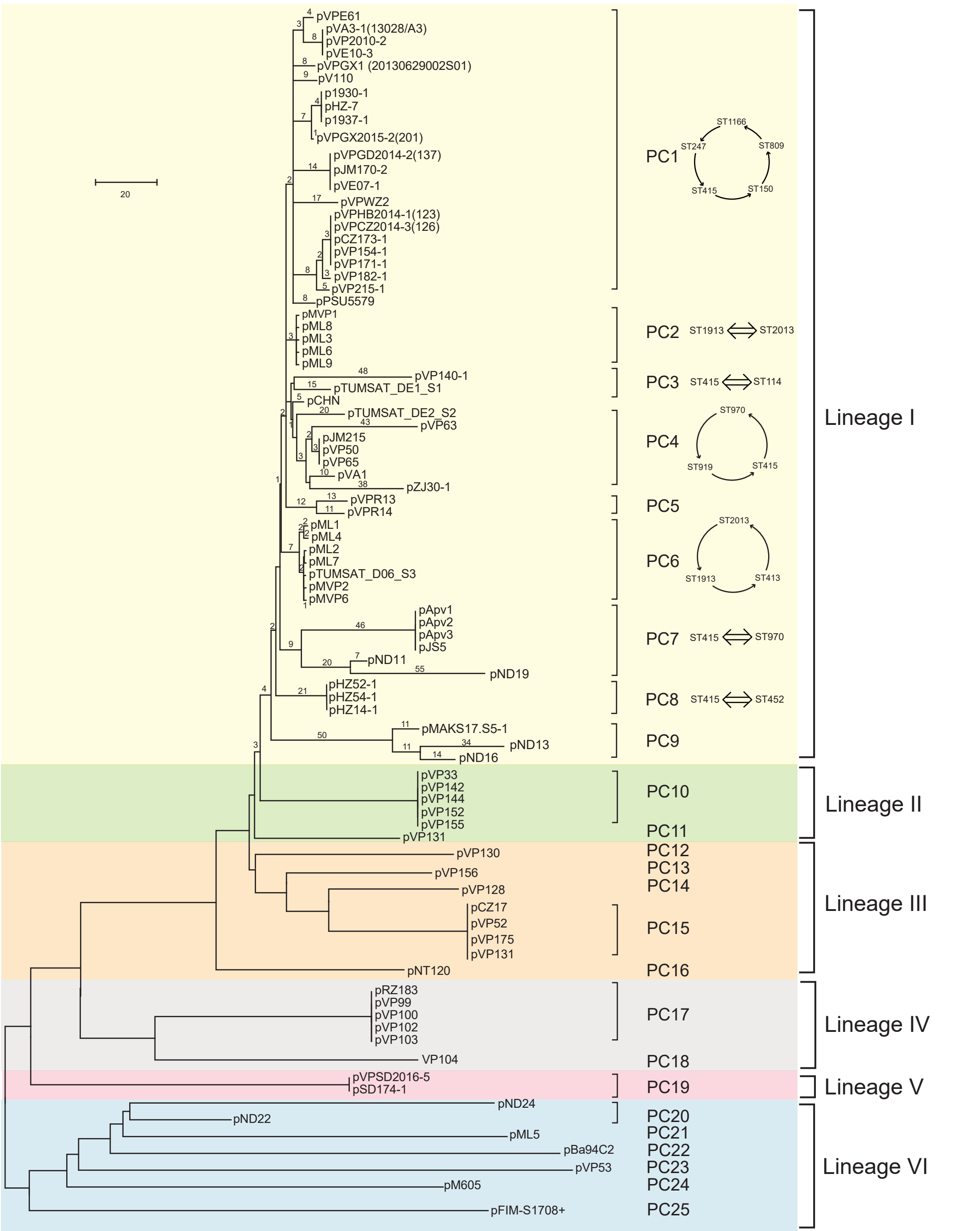

Supplement: FIG S3 [file mSystems.00799-19-sf003.pdf]

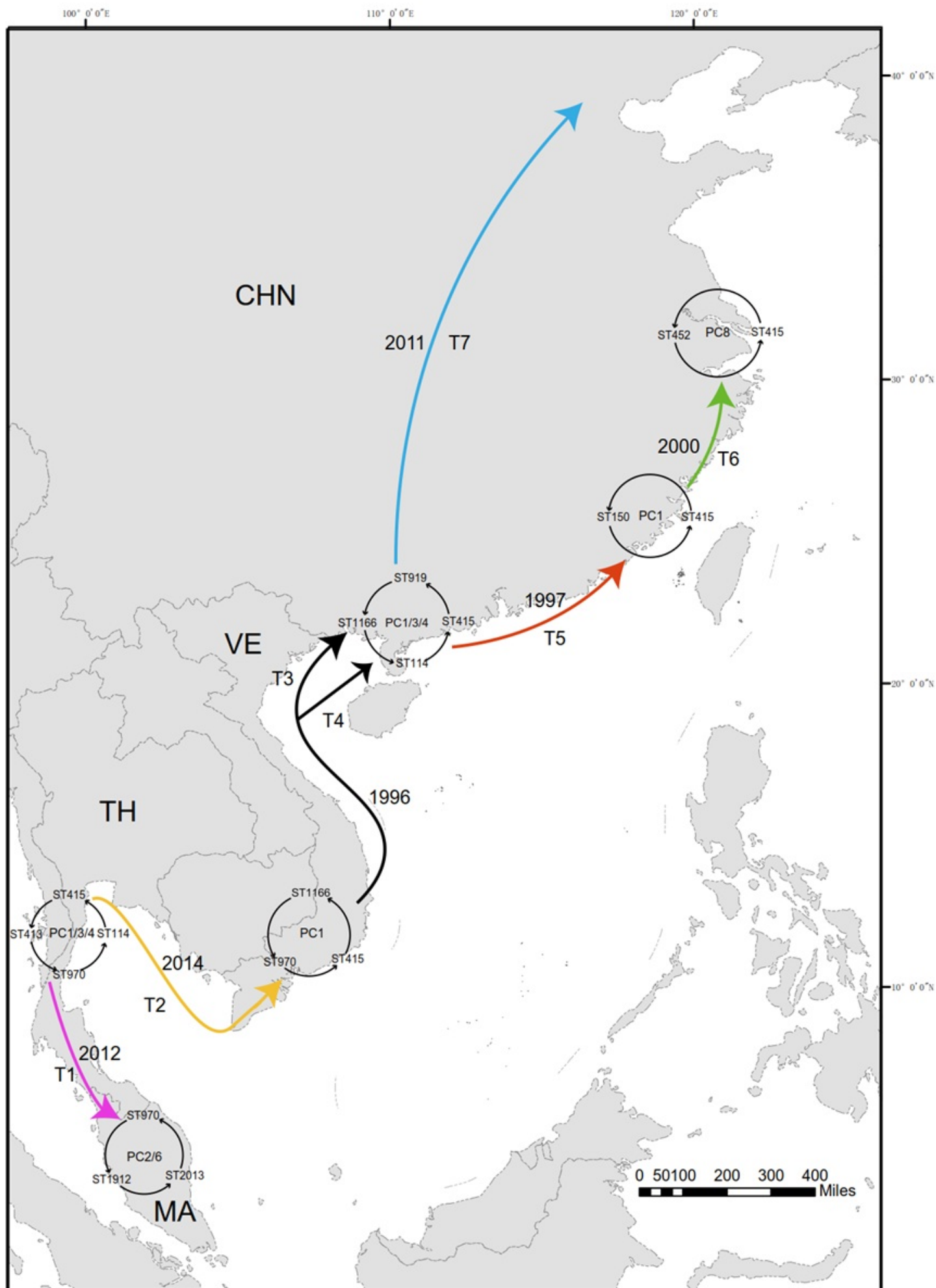

Supplement: FIG S4 [file mSystems.00799-19-sf004.pdf]

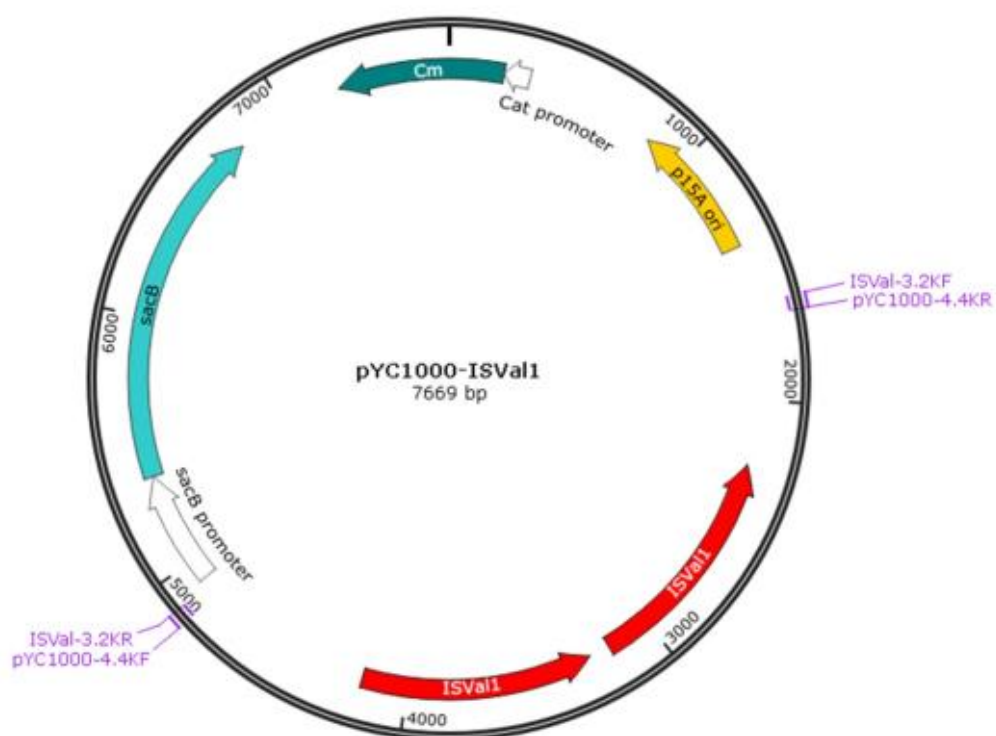

Supplement: FIG S5 [file mSystems.00799-19-sf005.pdf]
