# Supplementary material for: Horizontal Plasmid Transfer Promotes the Dissemination of Asian Acute Hepatopancreatic Necrosis Disease and Provides a Novel Mechanism for Genetic Exchange and Environmental Adaptation
Source: mSystems. 2020 Mar 17;5(2):e00799-19. doi: 10.1128/mSystems.00799-19 (PMC7380584; doi:10.1128/mSystems.00799-19)
Supplement: TEXT S1 [file mSystems.00799-19-s0001.docx]

**Supplementary text1**

**Phylogentic analysis of *pirAB*-positive plasmid**

Phylogenetic analysis of 25 *pirAB*-positive plasmids from Thailand and Malaysia revealed that seven plasmids from ST970 and ST390 were distributed into six distant clusters, while remaining plasmids from four STs (ST970, ST114, ST1913 and ST2013) were grouped together. These results suggested that some STs of *Vp*_AHPND_ originated from Thailand and spread into Vietnam and Malaysia after 2010. Thereafter, horizontal transfer of plasmids among different STs promoted the dissemination of AHPND endemically.

To obtain an overview of historical plasmid transfer events accounting for the dissemination of AHPND, we assembled the *pirAB*-positive plasmid from *Vp*_AHPND_ genomes used in this dataset. Only 88 plasmids with a size of over 50 kb were selected. We retrieved 6,437 SNPs from the plasmids by using pVA-1 as reference. A previously defined core sequence of *pirAB*-positive plasmid was used to construct a Maximum-parsimony (MP) tree. A plasmid MP tree was then constructed, which divided 88 *pirAB*-positive plasmids into six lineages; 64.5% of the plasmids belonged to Lineage I (Figure S3). Plasmids from ST1743, from ST424, and from ST809 formed Lineages II, III, and VI, respectively. Two plasmids from ST150 formed Lineage V. Plasmids in Lineage VI came from multiple STs from SE-Asia, China, and America.

However, a closer look at Lineage I revealed that horizontal plasmid transfer events might have occurred extensively among different STs. To better identify the possible plasmid transfer events, we defined the plasmid clone (PC) by taking 105 SNPs difference among the plasmids as a cutoff value (based on in-field observation from Dong et al. (2017) and this study). Thus, 25 PCs were identified, of which 9 PCs (PC1 to PC9) belonged to Lineage I. PC1 is the largest plasmid group, which comprised plasmids from six different STs (ST1166, ST809, ST415, ST150). The dissemination of these plasmids might associate with the movement of ST415 and ST1166 by ocean currents from the Thailand Gulf to the estuary of the Mekong River and subsequent movement to the coastal line in South China (Figure S4). The PC2 and PC6 come from Southeast-Asia; potential plasmid transfer events were also identified between Thailand and Malaysia as described above. The PC3 and PC4 contain reference plasmid pVA-1 and other plasmids from Thailand and China that came from four STs. PC7 included four plasmids from ST415 and two plasmids from ST970, while PC8 consisted of *pirAB*-positive plasmids from ST415 and ST452 as confirmed above. Compared with other PCs in Lineage I, PC5 (from an unknown ST in Pacific Ocean) and PC9 (from ST970 in Malaysia) have considerable sequence divergences. However, it is still reasonable to speculate that PC1 to PC9 might all originate from a common ancestor.

Overall, the high similarity of plasmids in Lineage I was likely caused by the transmission of ST1166, ST970, and ST415. However, the clustering of the remaining plasmids (PC10 to PC25) also reflected a high genetic diversity. A possible explanation is that these plasmids was acquired long-before 2010 and became endemically in the local environment_._
